# The role of the Hippo pathway in the pathogenesis of inflammatory bowel disease

**DOI:** 10.1038/s41419-021-03395-3

**Published:** 2021-01-12

**Authors:** Zhuo Xie, Ying Wang, Guang Yang, Jing Han, Liguo Zhu, Li Li, Shenghong Zhang

**Affiliations:** Division of Gastroenterology, The First Affiliated Hospital, Sun Yat-sen University, Guangzhou, People’s Republic of China

**Keywords:** Post-translational modifications, Chronic inflammation

## Abstract

Inflammatory bowel disease (IBD) is a chronic and recurrent inflammatory disorder that primarily comprises Crohn’s disease (CD) and ulcerative colitis (UC). Owing to its increasing prevalence in Eastern countries and the intractable challenges faced during IBD treatment, extensive research on IBD has been carried out over the last few years. Although the precise aetiology of IBD is undefined, the currently accepted hypothesis for IBD pathogenesis considers it to be a combination of environment, genetic predisposition, gut microbiota, and abnormal immunity. A recently emerged signalling pathway, the Hippo pathway, acts as a key regulator of cell growth, tissue homoeostasis, organ size, and has been implicated in several human cancers. In the past few years, studies have revealed the importance of the Hippo pathway in gastrointestinal tract physiology and gastrointestinal diseases, such as colorectal cancer and IBD. However, the role of the Hippo pathway and its exact impact in IBD remains to be elucidated. This review summarises the latest scientific literature on the involvement of this pathway in IBD from the following perspectives that account for the IBD pathogenesis: intestinal epithelial cell regeneration, immune regulation, gut microbiota, and angiogenesis. A comprehensive understanding of the specific role of the Hippo pathway in IBD will provide novel insights into future research directions and clinical implications of the Hippo pathway.

## Facts

- Inflammatory bowel disease (IBD) is a chronic intestinal disorder that severely influences the quality of life of patients, and its precise aetiology remains to be defined.
- The Hippo pathway is a highly conserved kinase cascade that regulates cell proliferation, survival, mobility, stemness, and differentiation. Studies suggest the regulation of intestinal diseases by the Hippo pathway, such as colorectal cancer and IBD but its role and impact in IBD remains unknown.
- The findings regarding the role of the Hippo pathway in IBDs may point out future research directions and provide novel insights into developing novel therapeutic approaches for treating IBD.
- A comprehensive understanding of the interplay between the Hippo pathway and IBD pathogenesis will help to assess the effect and feasibility of targeting molecules of the Hippo pathway in IBD.

## Open questions

- What’s the specific role of the Hippo pathway in regulating the inflammation and immune response associated with IBD?
- Whether targeting Hippo pathway will promote regeneration of intestinal epithelium and have positive implications on the treatment of patients with IBD.
- In addition to YAP in macrophages, will YAP in other cell types and other components of the Hippo pathway affect the gut microbiota?
- What’s the precise function of the Hippo pathway in the angiogenesis of IBD?

## Introduction

Inflammatory bowel diseases (IBDs) are incurable inflammatory disorders that severely influences the quality of life of patients^1^. With the increasing incidence of IBD, the burden on the global economy has become considerable^2^. Hence, there is a need to consolidate scientific knowledge in order to develop effective therapeutic methods to treat IBD. Despite the incomplete aetiology of IBD pathogenesis, available experimental and clinical evidence suggests that the genesis, development, and outcomes of IBD are related to the environment, genetic predisposition, intestinal micro-ecology, and immunological imbalance.

Intriguingly, the Hippo pathway, a common pathway involved in the tumour signalling, has recently drawn attention of researchers. The Hippo pathway is an evolutionarily conserved pathway that controls organ size and homoeostasis through modulating cell proliferation, survival, apoptosis, and stemness^3^. It comprises a cascade of kinases, including the mammalian Ste20-like kinases1/2 (MST1/2), adaptor protein salvador 1 (SAV1), large tumour suppressor 1/2 (LATS1/2), and Mps one binder 1 (MOB1); the yes-associated protein (YAP) and transcriptional co-activator with PDZ-binding motif (TAZ) are the major effectors^4^. Recently, studies have identified that nuclear Dbf2-related 1/2 (NDR1/2)^5,6^ and mitogen-activated protein kinase kinase kinase kinases (MAP4Ks)^7^ are novel members of the Hippo pathway. It has been suggested that the Hippo pathway is involved in intestinal inflammation diseases such as the inflammatory bowel disease (IBD)^8,9^. However, the role played by Hippo pathway in the pathogenesis of IBD remains unclear.

Herein, we summarise the updated scientific knowledge regarding the Hippo pathway and its role in the pathogenesis of IBDs, mainly from the following aspects: intestinal epithelial cell regeneration, gut microbiota, and angiogenesis. We also discuss future research directions and potential therapeutic approaches for treating IBD based on these findings.

## The Hippo pathway

### The components of the Hippo pathway

The Hippo pathway is a highly conserved kinase cascade that primarily regulates cell proliferation, survival, mobility, stemness, and differentiation, and was originally discovered in *Drosophila melanogaster* via genetic screens of tumour suppressors whose loss of function may lead to tissue over growth. The mammalian core kinases of the Hippo pathway comprise MST1/2 (ortholog of *Drosophila* Hippo/Hpo)^10^, SAV1 (also called WW45, salvador ortholog), LATS1/2 (Warts/Wts ortholog)^11,12^, and MOB1A/B (Mats ortholog)^13^. In addition, NDR1/2 (Trc ortholog)^14^ and MAP4K (Happyhour/Hppy ortholog)^15^ have been recently identified as novel components of the Hippo pathway^14^. The transcriptional co-activators YAP (also known as YAP1, Yorkie/Yki ortholog)^16^ and TAZ (YAP paralog in mammals)^17^ are the primary downstream effectors of the Hippo pathway^18^. The main binding partners of YAP/TAZ are transcriptionally enhanced associated domains (TEADs, scalloped/Sd ortholog)^19,20^, which bind to DNA to initiate transcription.

### Upstream signals dependent on the Hippo pathway

**Fig. 1:**
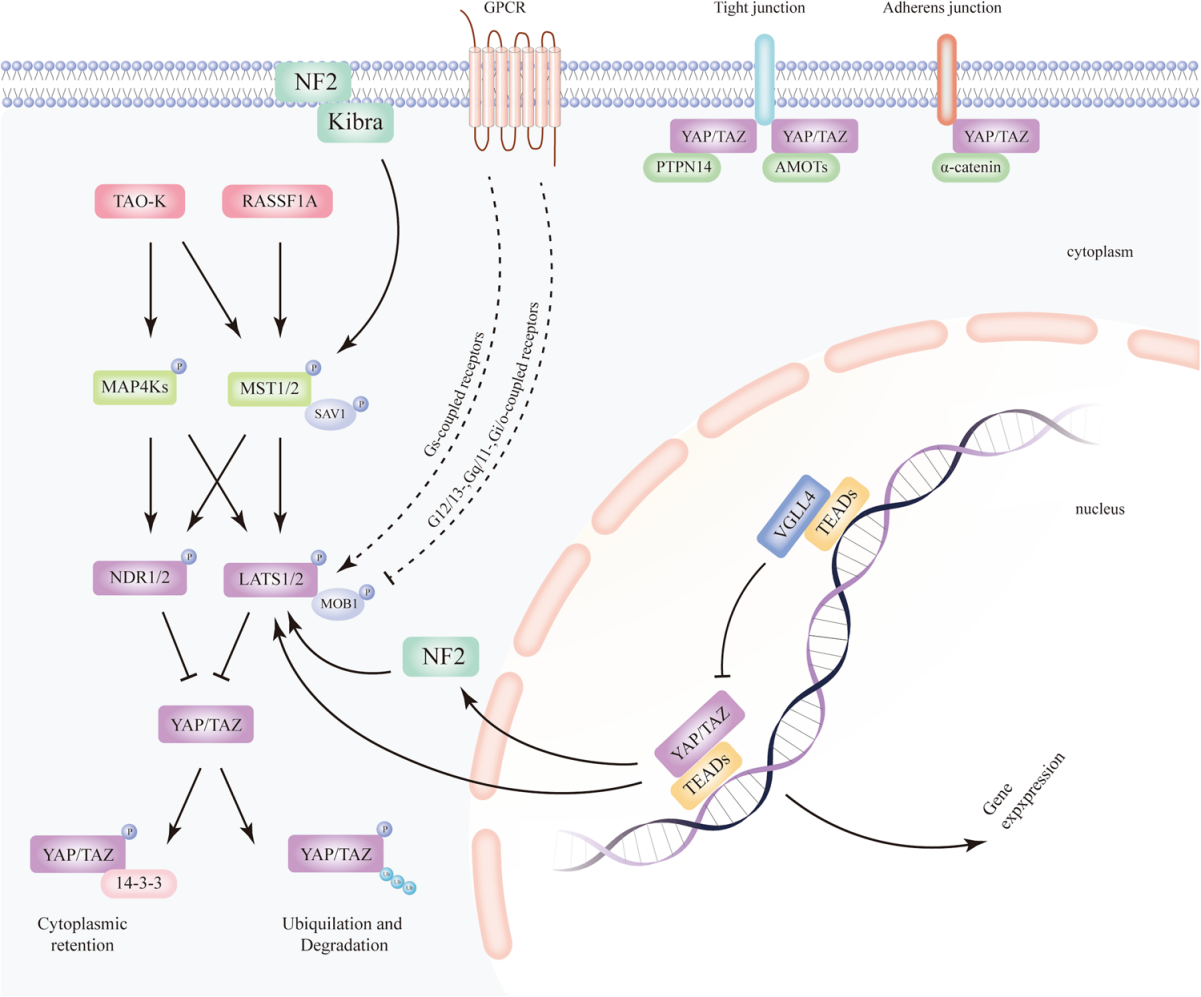
Regulation of the mammalian Hippo pathway. The core components of the Hippo pathway encompass MST1/2 and LATS1/2, whereas YAP/TAZ are two major effectors of this pathway. Traditionally, upstream signals turn on the Hippo pathway by phosphorylating and activating MST1/2 as well as the scaffold protein SAV1, followed by phosphorylation and activation of LATS1/2 and the scaffold protein MOB1, thereby preventing YAP/TAZ from translocating to the nucleus and initiating gene expression via binding to TEADs. VGLL4, a transcriptional co-repressor, competes with YAP/TAZ for TEADs binding. Instead, phosphorylated YAP/TAZ are isolated in the cytoplasm through interaction with protein 14-3-3 or experience ubiquitylation and degradation. Expanded Hippo pathway encompasses MAP4Ks as well as NDR1/2. MAP4Ks function similarly as MST1/2, performing activating phosphorylation of LATS1/2 and/or NDR1/2, consequently leading to the inhibition of downstream YAP/TAZ via LATS1/2- or NDR1/2-regulated phosphorylation. The complex of YAP/TAZ and TEADs can both directly enhance the function of LATS2 and indirectly stimulate LATS1/2 expression via NF2, which constitute a feedback loop. Independent of the upstream kinases, YAP/TAZ may be directly sequestered at cell junctions via interacting with PTPN14, AMOTs, and α-catenin. NF2, Kibra, RASSF1A, and TAO-K stimulated by upstream stimuli, may phosphorylate and activate MST1/2 and/or MAP4Ks. GPCRs may inhibit and enhance LATS1/2 depending on the types of receptors. Arrows, blunt ends, and dotted lines indicate activation, inhibition, and possible regulatory effect, respectively. MST1/2, mammalian sterile 20-like 1/2; SAV1, salvador; LATS1/2, large tumour suppressor homologue 1/2; MOB1A/B, MOB kinase activator 1A/B; YAP, yes-associated protein; TAZ, transcriptional co-activator with PDZ-binding motif; NF2, neurofibromin2/Merlin; TAO-K, thousand and one amino acid protein kinase; MAP4Ks, mitogen-activated protein kinase kinase kinase kinase; TEAD, transcriptional enhancer associated domain; VGLL4, vestigial-like family member 4; PTPN14, protein tyrosine phosphatase non-receptor type 14; AMOT, angiomotin.

Multiple cell signals, such as cell polarity, cell adhesion, cell–cell contact, stress, mechanical cues and hormones can stimulate the Hippo pathway^3,21,22^. Neurofibromatosis type II/Merlin (NF2) and Kibra can activate MST1/2, whereas thousand and one amino acid protein kinase (TAO-k) has been shown to activate MST1/2 and MAP4Ks^23–25^. Ras association domain family protein 1 A (RASSF1A) has been shown to facilitate the activation of the Hippo kinase MST1/2^26^, thereby promoting apoptosis^27,28^. G-protein-coupled receptor (GPCR) signalling can either activate or inhibit LATS1/2 indirectly, depending on the coupled G-protein^29^. LATS1/2 kinases can be suppressed via signal transduction mediated by G12/13-, Gq/11-, and Gi/o-coupled receptors, thereby activating the function of YAP/TAZ. In contrast, stimulation of Gs-coupled receptors leads to the activation of LATS1/2 and inhibition of its downstream effectors YAP/TAZ. MAP4Ks can activate LATS1/2 and NDR1/2 in the Hippo pathway, acting in parallel with MST1/2^7^. MAP4Ks can directly activate LATS1/2 as alternative MST1/2-like kinases^30^. Studies have shown that NDR1/2 is a novel member of the Hippo pathway^14^. NDR1/2 acts downstream of MST1/2 and MAP4K, and subsequently inhibits YAP/TAZ signalling in parallel to LATS1/2^5^ (Fig. 1).

### Upstream signals independent of the Hippo pathway

In addition to activating the core Hippo kinases to inhibit YAP/TAZ, upstream signals can also directly suppress nuclear localisation of YAP/TAZ. YAP/TAZ can be sequestered at tight junctions via conjugation of the angiomotin family of proteins (AMOT) and protein tyrosine phosphatase non-receptor type 14 (PTPN14) or at adherens junctions via interaction with α-catenin^23^ (Fig. 1).

### The regulatory mechanism of the Hippo pathway

Once the Hippo pathway is turned on by upstream signals, Hippo kinases MST1/2 and/or MAP4Ks are phosphorylated and activated, which in turn activate LATS1/2, MOB1, and/or NDR1/2^31^. Subsequently, YAP and TAZ are phosphorylated, leading to sequestering of YAP/TAZ in the cytoplasm through interacting with 14-3-3 proteins or via poly-ubiquitination mediated proteasomal degradation^32,33^. In contrast, when Hippo signalling is inhibited, MST1/2 and/or MAP4Ks lose their activity and function, LATS1/2 and NDR1/2 are dephosphorylated, thereby dephosphorylating YAP/TAZ. Dephosphorylated YAP/TAZ subsequently translocate to and accumulate in the nucleus where they bind to DNA-binding TEADs and other transcription factors^34^, initiating target gene transcription to induce cell proliferation, differentiation, death, survival, and stemness, and thus, regulating tissue homoeostasis and growth^35–37^.

Nevertheless, in the absence of the nuclear YAP/TAZ, TEADs bind to the vestigial-like family 4 (VGLL4, Tgi ortholog)^38^ proteins, but are unable to bind to the target YAP/TAZ genes. This leads to the inhibition of expression of genes regulated by YAP/TAZ^3,4,11,39^ (Fig. 1).

### A negative feedback loop of the Hippo pathway

As mentioned above, LATS1/2 act as negative regulators of YAP/TAZ. Moroishi et al.^40^ showed that the complex of YAP/TAZ and TEADs can both directly stimulate the expression of LATS2 kinases and indirectly induce LATS1/2 expression via NF2, which constitute a negative feedback of the Hippo pathway (Fig. 1). Tissue development and regeneration can be promoted by hyperactivation of YAP/TAZ, whereas it is impaired by their inactivation^39,41,42^. Thus, this feedback loop is an efficient mechanism to regulate YAP/TAZ homoeostasis and function^40^.

### Interplays between the Hippo signalling and other pathways

The Hippo pathway does not work alone. Increasing research in recent years has indicated an interplay between the Hippo and other pathways in intestinal regeneration, including the WNT^43^ and Notch^44^ pathway. Furthermore, the Hippo pathway has been suggested to be associated with inflammation, including the NF-κB pathways^45,46^ and other components^9,47^, and also with immune-regulating pathways^15^.

#### Hippo and WNT pathways

WNT signalling plays an essential role in intestinal tissue homoeostasis and stem cell maintenance. β-catenin is a major effector of the WNT signalling pathway. WNT ligands release β-catenin from proteasomal degradation via conjugation with Frizzled (FZ) and low-density lipoprotein receptor-related protein (LRP) receptors, contributing to the accumulation of β-catenin and initiation of Dishevelled (DVL). Nuclear β-catenin binding to TCF initiates WNT target gene expression with the assistance of DVL^48,49^. Stimulation of WNT signalling, represented by β-catenin translocating to the nucleus, enhances the differentiation of Paneth cells at the base of the crypt^50^. Conversely, inhibition of WNT signalling via disruption of the T-cell factor7/2(Tcf7/2 gene), which codes Tcf-4 in mice, causes loss of transit-amplifying cells (TAC) and deeply impairs the crypt structure, thereby affecting the normal structure of the gut^51^.

**Fig. 2:**
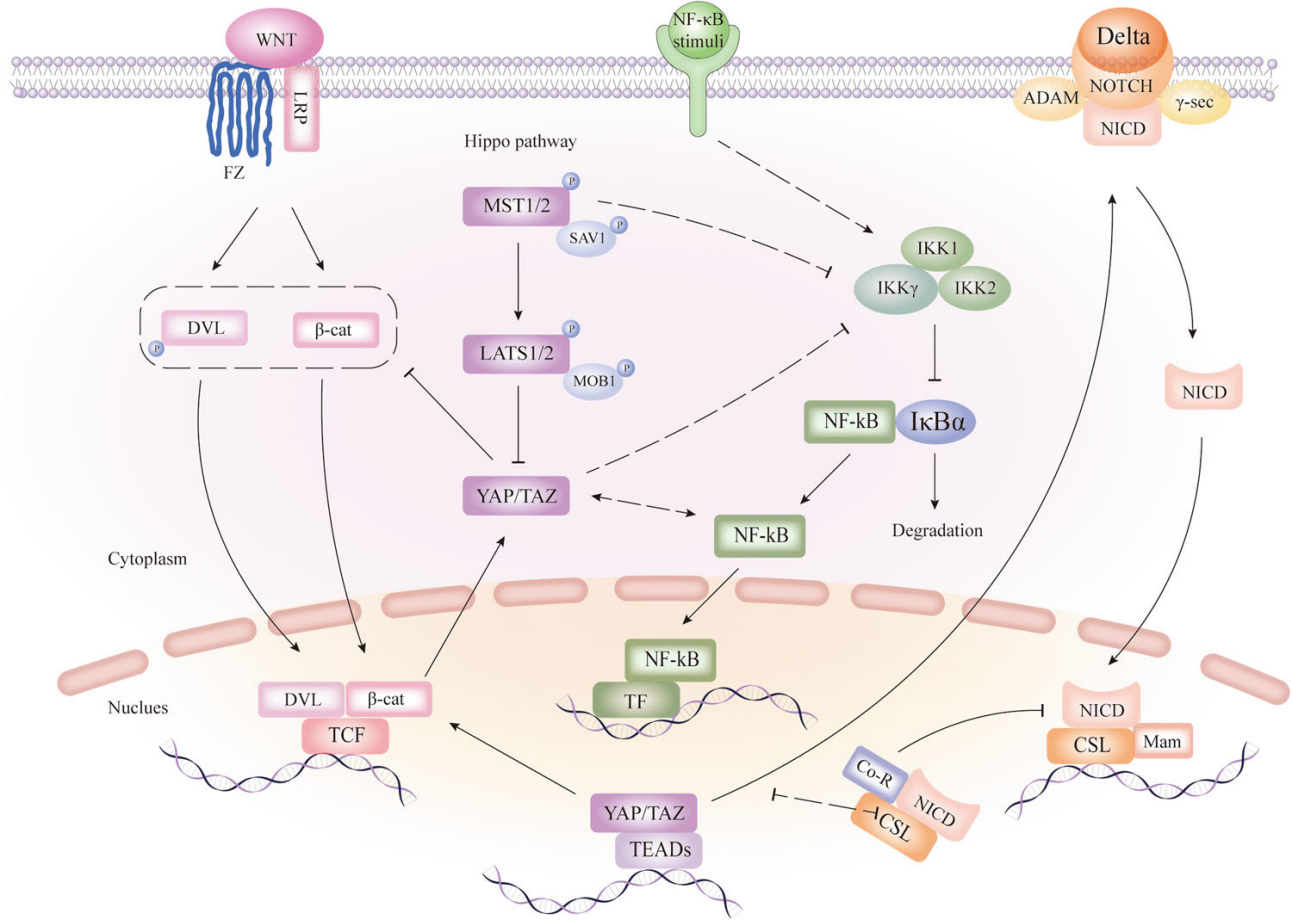
The mutual interaction of WNT, Notch, and Hippo pathway in the intestine. Cytoplasmic YAP/TAZ inhibits WNT target gene expression via DVL phosphorylation and β-catenin activation, whereas nuclear YAP promotes WNT signalling by cooperating with β-catenin. YAP/TAZ may also be activated by activated β-catenin. MST1 and YAP can indirectly inhibit the regulatory subunit IKKγ, leading to the inhibition of NF-κB. Besides, YAP and NF-κB can both inhibit and activate each other indirectly in different situations. In addition, YAP/TAZ may activate Notch signalling through transcriptional regulation. Arrows and blunt ends indicate activation and repression, respectively. The dotted line represents the indirect regulation. Fz, frizzled; LRP, low-density lipoprotein receptor-related protein; DVL, dishevelled; β-cat, β-catenin; TCF, T-cell factor; MST1/2, mammalian sterile 20-like 1/2; SAV1, salvador; LATS1/2, large tumour suppressor homologue 1/2; MOB1A/B, MOB kinase activator 1A/B; YAP, yes-associated protein; TAZ, transcriptional co-activator with PDZ-binding motif; TEAD, TEA domain family member; NF-κB, nuclear factor NF-κB; IKK, IκB kinase; IκBα, NF-κB inhibitor; ADAM, ADAM-family metalloproteases; γ-sec, γ-secretase; NICD, Notch intracellular domain; CSL, CBF1, Su(H) and LAG-1; Co-R, co-repressor; Mam, mastermind.

Precedent studies have shown that the regulation of WNT signalling by YAP/TAZ depends on the phosphorylation state and cellular localisation of YAP/TAZ proteins. Cytoplasmic YAP/TAZ hinders WNT signalling by repressing DVL phosphorylation and restricting nuclear translocation and activation of β-catenin^52,53^. In contrast, nuclear YAP interacts with and stabilises β-catenin, thus increasing the expression of WNT target genes^54,55^. In summary, the activation of the Hippo pathway inhibits the WNT signalling pathway via cytoplasmic and phosphorylated YAP/TAZ, whereas shutdown of the Hippo pathway facilitates WNT target gene expression by nuclear and dephosphorylated YAP. In addition, WNT-activating β-catenin has been reported to activate and up-regulate YAP and TAZ^56,57^. Mechanistically, YAP expression is driven by the binding of β-catenin/TCF4 complexes to the first intron of the YAP gene that enhances the transcription of DNA^56^. Phosphorylation of β-catenin leads to the degradation of TAZ, while depletion of β-catenin impairs TAZ/β-TrCP interaction^57^ (Fig. 2).

#### Hippo and Notch pathways

Initiated by the interaction of the Delta ligand on one cell with the notch receptor on a different cell generates two proteolytic cleavage events, which are catalysed by the ADAM-family metalloproteases and γ-secretase, leading to the translocation of Notch intracellular domain (NICD) to the nucleus and its subsequent interaction with DNA-binding CSL (CBF1, Su(H), and LAG-1) and co-activator Mam (mastermind) proteins. Simultaneously release of co-repressors (Co-R), which repress target gene expression, further activates Notch signalling^58,59^.

Reciprocally, the Hippo pathway, as reported in previous studies, can modulate Notch signalling. Conditional knockout of MST1/2 specifically in the intestine promotes nuclear accumulation of NICD. Depletion of MST1/2 strongly activates Notch signalling via decreased phosphorylation, increased abundance, and nuclear accumulation of YAP^44^. Intrinsically, the nuclear YAP facilitates Notch signalling^60^. Therefore, the Hippo pathway can inhibit Notch signalling by phosphorylating and repressing YAP (Fig. 2).

#### Hippo and pathways involved in inflammation

##### Hippo and NF-κB pathways

The nuclear factor (NF)-κB signalling pathway has been widely recognised as a key role in the regulation of inflammation^61^. The NF-κB family can be activated through canonical and non-canonical signalling pathways^62^. The “canonical” pathway is triggered by multiple stimuli including proinflammatory cytokines^63^, which function as activators of the canonical IκB kinase (IKK) complex, including the kinase subunits IKKα (IKK1), IKKβ (IKK2) and the regulatory subunit IκB kinase γ (IKKγ), also called NEMO. The activated IKK complex mediates the phosphorylation, ubiquitination and proteasomal degradation of the NF-κB inhibitor IκBα, with NF-κB subsequently released from the suppression of IκBα in the cytoplasm where it then translocates to the nucleus, leading to the target genes transcription^64^.

A recent study uncovered that MST1 inhibits the NF-κB signalling stimulated by tumour necrosis factor-α (TNF-α) in the inflammatory response^46^. Mechanistically, MST1 phosphorylates the catalytic component of the E3 ligase linear ubiquitin assembly complex (LUBAC), namely HOIP, and thus suppresses linear ubiquitination of the regulatory subunit NEMO/ IKKγ, and thereby attenuates the activation of the NF-κB signalling pathway depending on LUBAC^46^.

It is worth noting that the reciprocal regulation between YAP/TAZ and NF-κB is sophisticated. A reciprocal antagonistic relationship between YAP/TAZ and NF-κB was reported in the regulation of osteoarthritic cartilage degradation^65^. YAP was reported to suppress the activation of NF-κB signalling via promoting the degradation of tumour necrosis factor receptor-associated factor 6 (TRAF6) in modulating endothelial activation and vascular inflammation^66^. Reversely, it was observed that YAP and NF-κB pathways activate each other reciprocally in human colon cancer cells^67^. In addition, a recent study also showed that YAP activates NF-κB activity in soft tissue sarcomas through suppressing a negative regulator of NF-κB signalling, namely USP31^45^. Collectively, YAP and NF-κB can both inhibit and activate each other indirectly under different situations (Fig. 2).

##### Hippo and other components involved in inflammation

With increasing research on the Hippo signalling pathway, studies in recent years have revealed that the Hippo signalling pathway is also involved in the occurrence and development of inflammation^68^. Inflammation is a complicated process with multiple mechanisms^69^ and a basic protective response^70^, but can transform into the primary contributor of the pathogenesis of many chronic diseases. It was pointed out that the Hippo pathway can be strongly activated by Gram-positive bacteria, leading to a decrease of antimicrobial peptides secretion and restriction of inflammation in drosophila^71^. The Hippo pathway key effectors, YAP and/or TAZ, were reported to be mediators or regulators of many inflammatory processes^72–74^. Furthermore, the role of other components of the Hippo pathway, including NDR1/2^75^ and MST1^76^ in inflammation has been emerging within recent years.

A prototypical proinflammatory cytokine, IL-6^77^, was found to potently activate YAP and Notch by binding its co-receptor gp130^9^. In addition, the expression of YAP target genes, including connective tissue growth factor (CTGF) were also observed to be upregulated in gp130 transgenic mice^9,19^.

#### Hippo and pathways involved in immune regulation

In addition to regulating inflammation, the Hippo pathway has been recently shown to play a role in immune modulation^78^.

MST1 plays a key role in mediating T-cell migration, adherence, and survival via its downstream effectors LATS1/2, NDR1/2, and YAP^79^. MST1 enhances regulatory T-cell (Treg) function via modulating Foxp3 acetylation^80^ with deficiency of MST1 having been shown to impair Foxp3 expression and Treg cell development and function in mice^81^. Nehme et al.^82^ also reported that MST1 deficiency may lead to the loss of naïve T cells, thereby influencing the autoimmune manifestation and causing recurrent bacterial or viral infections^82^. A novel primary immunodeficiency syndrome was described to be caused by the STK4 (namely MST1) mutation and deficiency, which affects lymphocytes and possibly neutrophil granulocytes^83^.

Furthermore, Jing et al.^84^ found that TAZ can function as a critical determinant of proinflammatory T helper (Th) 17 cells and immunosuppressive Treg cells. More specifically, lack of TAZ promotes Treg cell differentiation, whereas TAZ transgenic expression or TAZ activation enhances Th17 cell differentiation. Interestingly, TEAD1 antagonises the effect of TAZ, thus inhibiting the differentiation and development of Th17.

Regarding IBD immunopathogenesis, recent advances have demonstrated that the innate immunity, which includes the mucus layer, antimicrobial peptides, and autophagy, plays an equally important role in inducing gut inflammation as adaptive immunity, which involves Th1, Th2, and other T cells, including Th17 and Treg cells^85^. The imbalance of Treg and Th17 cells contributes to the inflammatory disease development^84^.

**Fig. 3:**
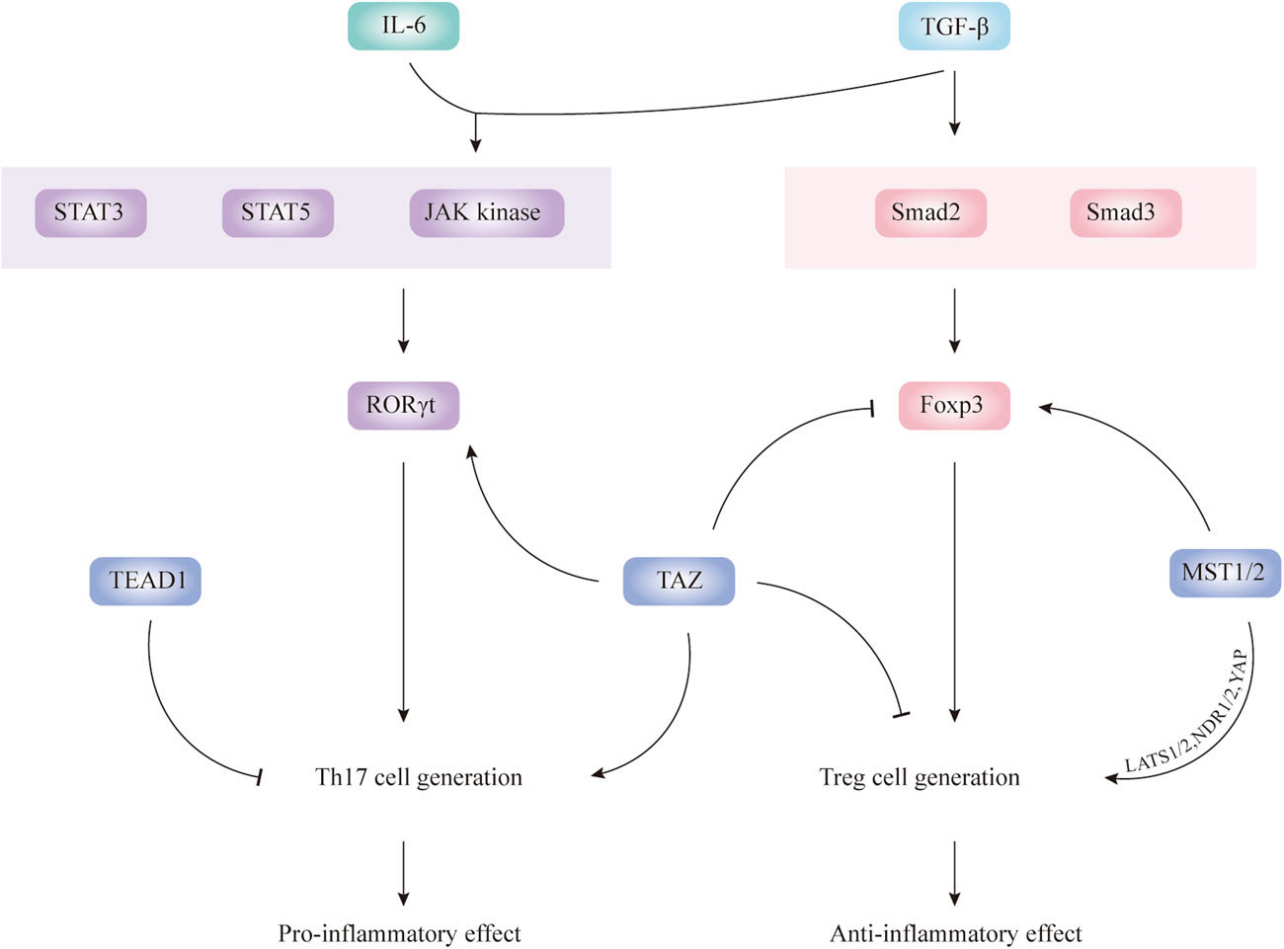
Hippo pathway and immune inflammation. TGF-β stimulates Smad2 and Smad3, thereby activating Foxp3 and promoting Treg cell differentiation. TGF-β and IL-6 together stimulate JAK kinases and transcription factors STAT3 and STAT5, subsequently activating RORγt and Th17 cell generation. MST1/2 enhances the stability of Foxp3 and promotes Treg cell generation through LATS1/2, NDR1/2, and YAP. In contrast, TAZ inhibits foxp3 and attenuates Treg cell generation but promotes the process of Th17 cell generation. In addition, TEAD1 may hinder Th17 cell formation. Arrows and blunt ends indicate activation and repression, respectively.

Transforming growth factor-beta (TGF-β) alone stimulates Smad2 and Smad3, thereby activating Foxp3 and promoting Treg cell differentiation. TGF-β and interleukin 6 (IL-6) together stimulate JAK kinases and transcription factors STAT3 and STAT5, subsequently activating RORγt and Th17 cell generation^86^. Th17 cells are characterised by the secretion of the signature cytokine IL-17A; the IL-17a transcript levels tested in CD and UC intestinal mucosa are comparatively higher than those in the control group^87,88^. Th17 cells promote inflammation in IBD. Conversely, Foxp3^+^ Treg cells stimulated by TGF-β alone display potent immunosuppressive effects in experimental colitis, and are increased in the IBD gut mucosa^89,90^ (Fig. 3).

Generally, M1 macrophages along with their secreted proinflammatory cytokines, including IL-6, aggravate IBD, whereas M2 macrophages attenuate IBD severity^91,92^. Therefore, YAP in macrophages was reported to aggravate IBD^93^ as it hinders IL4/IL13 from inducing M2 polarisation of macrophages and enhances activation of M1 macrophages triggered by lipopolysaccharide (LPS)/interferon γ (IFN-γ).

### The intestine cellular structure and the role of Hippo pathway in mammalian intestine

The small intestine epithelium is single-layered with the crypts-villus tissue architecture^94^. The finger-like protrusion of the villi into the intestinal lumen enlarge the surface area and improve the absorptive capacity. Numerous capillaries and lymph vessels form underneath the epithelium of the villi help in absorbing nutrients. The crypts locate in invaginations of the intestinal wall. At the bottom of the crypts lie the intestinal stem cells (ISCs). The niche protecting the ISCs is formed by Paneth cells and the surrounding mesenchyme. ISCs differentiate into progenitor cells, also named transit-amplifying (TA) cells, which locate in the crypts above the ISCs^49^. TA cells will stay still if they retain the ability of stemness, if not, they will move towards the villi and differentiate into absorptive intestinal cells (such as enterocytes) and secretory cells (such as intestinal endocrine cells and goblet cells)^49,95^; or move towards the crypt bottom and differentiate into Paneth cells to nurture and protect the ISCs^96^. Whereas the epithelial surface of the large intestine is flat on account of lacking villi and Paneth cells; however, the colon has deep crypt secretory (DCS) cells, which serve as Paneth cell equivalents in the crypt niche and interact with Leucine-rich repeat-containing G-protein coupled receptor 5-positive (Lgr5(+)) stem cells at crypt bottoms^97^.

The activity of MST1/2 is lower in the crypts but higher as cells move from the crypts toward the lumen. Conversely, YAP is of high abundance in the nucleus of lower crypts regions, but also exists in upper TA cells and the villi, where it is primarily cytoplasmic^98^. As cells move from the crypts towards the villi, the nuclear expression of YAP is gradually decreased, whereas the cytoplasmic expression of YAP increases^44^. YAP is deficient in the small intestine but enriched in the nucleus of the colon. During normal intestinal mucosal turnover, YAP is generally restricted to the ISCs and is inactivated by the Hippo pathway, thereafter making no contribution to the proliferative capacity of ISCs and TA compartments^33^. However, ablation of MST1/2 from mouse intestinal epithelium results in enhanced abundance of nuclear YAP and subsequently the increased proliferation of undifferentiated ISCs and loss of secretory cells both of the small and large intestine^44^. In contrast to MST1/2, YAP/TAZ play a dual role in renewal of the intestinal epithelium, of which one is proliferating ISCs by collaborating with transcription factor TEADs, the other is promoting goblet cells differentiation via cooperating with transcription factor klf4^99^. Significantly, YAP is confirmed to promote tissue regeneration in mammalian intestine^8^.

Furthermore, evidence shows the connection of Hippo pathway with the intestinal epithelial junctions of mammalian. In mammalian intestine, cell–cell adhesion mediated via E-cadherin occurs at adherens junctions, where cell attachments are linked to the actin-myosin cytoskeleton by catenins and relevant proteins. Tight junctions locate in the apex of the adherens junctions, mammalian epithelial cells have tight junctions, which form a paracellular diffusion barrier^100^. Disruption of IEC polarity or epithelial junctions can activate the Hippo pathway and often causes intestinal diseases including IBD^101^. Upstream signals can modulate YAP/TAZ independent of the Hippo pathway via epithelial junctions^23^. Besides, E-cadherin-mediated cell–cell contact can control cell proliferation by regulating the Hippo signalling pathway^102^.

## The Hippo pathway and IBD

In the past few decades, many studies have focused on the function and regulation of the Hippo pathway in the gut epithelium^18,35,103^. However, the precise role of the Hippo pathway in IBDs remains largely unknown. Overviewing the role of Hippo pathway in IBD pathogenesis can helps us further understand the role played by this pathway in IBD pathogenesis, and provide insights into the potential therapeutic value of Hippo pathway targeting molecules in IBD treatment.

### Inflammatory bowel disease

IBD, clinically encompassing ulcerative colitis (UC) and Crohn’s disease (CD), is a chronic and recurrent inflammatory disorder of the gut. The incidence of IBD has dramatically increased worldwide over the past decades and has become a global challenge^2,104^. Extensive studies have been performed to understand the pathogenesis of IBD^105^. Although the precise cause of IBD remains unclear, the most commonly acceptable hypothesis for IBD pathogenesis implicates the interplay of genetic susceptibility, micro-ecology and immune imbalance^106^.

Colitis mouse models induced by dextran sulphate sodium (DSS) were used to investigate the role of several factors in the pathogenesis of IBD; trinitrobenzene sulphonic acid (TNBS) has been used to establish animal models to simulate the pathogenesis and clinical process of IBD^107–109^. DSS-induced colitis is triggered by the impairment of the epithelial barrier, which permits gut bacteria to penetrate the impaired mucosa and cause permanent mucosal inflammation. Enhanced inflammatory infiltration and overproduction of proinflammatory cytokines lead to the exacerbation of colitis^110^. Innate immune cells play a pivotal role in establishing DSS-induced colitis^111,112^, and TNBS, used as a hapten-modified self-antigen, induces an autoimmune response in mice^113,114^. Rectal TNBS administration in mice may elicit colonic mucosal immune response mediated by T cells. This results in persistent mucosal inflammation and simultaneous heavy infiltration of inflammatory cells in the mucosa and submucosa, and thus establishing the induction of colitis^109,115^.

### Hippo and the pathophysiology of IBD-associated colitis

#### Hippo and intestinal epithelial regeneration

The intestinal epithelium primarily encompasses absorptive (columnar epithelial) or secretory (Paneth, goblet, enteroendocrine, and tuft) intestinal epithelial cells (IECs)^116^, which function as a mucosal barrier to stop pathogenic bacteria from invading gut mucosa, thereby providing a favourable condition for the absorption of nutriens^117^. To confront and cope with the dynamic changes in the surrounding environment, IECs self-renew every 3-5 days. Once damaged, the intestinal epithelium experiences the complicated “epithelial restitution” process, which repairs the damaged epithelium, followed by intestinal stem cell (ISC) activation, proliferation, and differentiation^118^. Proper IEC regeneration supplements IBD treatment and complete intestinal mucosa regeneration in IBD patients represents long-term remission and a low risk of surgical treatment^119^. Therefore, the significance of IECs in IBD treatment has drawn considerable attention^120^.

Conditional knockout of MST1/2 in IECs leads to a disorganised villus structure, expansion of undifferentiated cells, and dysplastic epithelia^44^. SAV1 deficiency in mouse gut results in enlarged crypt structures^8^. Common secretory precursors or goblet cell progenitors regulated by the upstream kinases MST1/2 and LATS1/2 (the downstream transcriptional co-activators YAP/TAZ) may promote the differentiation of these cells into goblet cells through participation of a partner transcription factor, klf4^99^. Inactivation of YAP and/or TAZ is not significant under intestinal homoeostasis^60,121^, but severely impairs DSS-induced intestinal regeneration^8^. Activation of YAP/TAZ has been revealed to promote intestinal tissue repair and colonic regeneration in a mouse DSS colitis model via the extracellular matrix (ECM) remodelling and FAK/Src signalling activation, and ultimately reprograms the epithelium transiently into a primitive state, where the ISCs play an extremely important role^122^. Importantly, YAP/TAZ promote cellular proliferation via transcription of TEADs in the gut epithelium, especially in ISCs^47^. In particular, previous literature has reported that YAP is crucial for epithelial progenitor cell proliferation and differentiation, and is involved in epithelial repair^123^. YAP mRNA is significantly upregulated in IECs in patients with CD and mice with colitis^47^. In the DSS-induced colitis and regeneration models, YAP is overexpressed and colonic epithelial cells in crypts actively proliferate^8,43^. In patients with IBD as well as in the DSS-induced colitis mouse model, YAP regulates mucosal regeneration^9^. Overexpression of nuclear YAP in mice enhances IEC proliferation and mucosal regeneration^43^. In contrast, cytoplasmic YAP hinders the expansion of ISCs and vital components localised in the stem cell niche. Barry et al.^98^ demonstrated that in YAP transgenic mice, Paneth cells function as critical components of the ISC niche^96^ and are mis-localised and eventually disappear. It should be noted that YAP protein levels increase only during the early stages of regeneration but are restored to normal upon the complete repair of the intestinal structure^35^. To conclude, nuclear YAP plays a positive role during intestinal epithelium regeneration in IBD and may serve as a novel therapeutic target aiming at IBD^8,43^.

In addition, the crosstalk between the Hippo and the WNT pathway plays a crucial role in gastrointestinal tissue via modulating the proliferation of epithelial cells localised in the intestinal crypts^49^, maintaining homoeostasis, and promoting regeneration of gut epithelium in ISCs^124^. Deng et al.^43^ reported that YAP and β-catenin show growing nuclear localisation during regeneration following inflammation. Overexpression of nuclear YAP triggered WNT/β-catenin signalling and significantly improved the healing ability of IEC, thereby indicating that nuclear YAP promotes the proliferation of IEC via activating WNT/β-catenin signalling pathways. Moreover, during intestinal regeneration after tissue damage, cytoplasmic YAP inhibits WNT signals, disrupts the ISC niche, and limits the growth of stem and niche cells resulting abnormal migration of Paneth cells and decrease of ISCs. As a result, proliferative crypts are lost and intestinal regeneration is suppressed^98^. Yu and co-workers^124^ also showed that YAP plays a crucial role in the recovery of the intestinal epithelium after exposure to ionising radiation in mice. By restricting WNT signalling and over differentiation of Paneth cell, YAP transiently reprograms Lgr5^+^ ISCs, thereby promoting regeneration of IECs.

Notch signalling is involved in the differentiation and regeneration of intestinal epithelium and contributes to the maintenance of ISCs^125–127^. Hyperactivation of Notch facilitates the generation of absorptive cells, such as enterocytes, whereas the inhibition of Notch inhibits the properties of stemness and leads to differentiation into secretory lineage (such as goblet, enteroendocrine, and Paneth cells)^128,129^. In colitis inflamed mucosa, Notch signalling activation is observed in large numbers of IECs^127^. In addition, administration of gamma-secretase inhibitors (GSIs) that inhibit YAP-activating Notch^60^, leads to the induction or worsening of colitis^126^.

Overall, a delicate balance among WNT, Notch, and Hippo signalling pathways is involved in intestinal epithelial homoeostasis and regeneration, and disturbance of this balance impairs the regenerative ability of IECs

Taniguchi et al.^9^ demonstrated that activation of IL-6/gp130 signalling triggers YAP and Notch signalling in mice and human cells, resulting in the proliferation of IECs, thereby resisting mucosal impairment. Gp130 is a co-receptor for IL-6 cytokines, and its expression is increased in IBD^130^. Upon mucosal injury, IL-6 binds to gp130 in IECs and produces proinflammatory cytokines^131^. Later, the gp130-associated tyrosine kinases Src and Yes are stimulated to phosphorylate YAP, leading to the stabilisation and nuclear translocation of YAP, which promotes healing and maintenance of barrier function.

#### Hippo and gut microbiota homoeostasis

The gut microbiota refers to the complex and abundant microbial community present in the human gut, which is intimately related to the nutrition, gut homoeostasis, and immune responses of the host^132^. It is known to all that the micro-ecology plays a key role in the pathogenesis of IBD^133^. Particularly, the diversity and structure of the gut microbiota are of much significance in IBD. A reduced diversity of the gut microbiota activates and worsens the condition of IBD^134–136^. Imbalance between symbiotic microflora and opportunistic pathogens can also contribute to the deterioration of IBD^137^.

Zhou et al.^93^ found that in mice with DSS-induced colitis, eliminating YAP expression in macrophages increases the abundance of the IBD-attenuating gut microbiota (*Lactobacillus*, *Bacteroides*, and *Bifidobacterium*) and decreases gut flora associated with IBD deterioration (*Prevotella*, *β-Proteobacteria, γ-Proteobacteria, and Enterobacteriaceae*) compared with that of the control group^138–140^. Thus, it has been speculated that YAP-elimination in macrophages affects the diversity of the gut microbiota during the induction of IBD. Based on the previous reports, antimicrobial peptides, such as Ang4, Retn1b, and RegIIIγ have been reported to promote the homoeostasis of gut microbiota, and their expression is speculated to increase via depletion of macrophages expressing YAP after IBD induction^93^.

Collectively, YAP expression in macrophages disrupts the gut homoeostasis and may alter the gut microbiota that contributes to the pathological process of IBD.

#### Hippo signalling and angiogenesis

Angiogenesis is the process of forming new vessels from pre-existing vasculature, and consists of several steps including vascular endothelial cell (EC) proliferation, migration, differentiation, lumen formation, and maturation, resulting in the expansion of the microvascular bed^141^. Not only does angiogenesis participate in physiological conditions such as tissue development, but it is also pivotal in many pathological conditions, including IBD^142^. Pathological angiogenesis is stimulated by inflammation and is impacted by the immune response^141^. Growing evidence has revealed intense angiogenesis in patients with UC and CD as well as in experimental colitis^142,143^, and that angiogenesis is an integral component of the pathophysiology of IBD^144,145^.

During the last few years, studies based on several model organisms have indicated that Hippo pathway plays a role in angiogenesis^146^. The Hippo pathway is currently identified as a critical regulator of EC proliferation, migration, and survival; hence, it is believed to participate in sprouting, barrier formation, and remodelling of the vascular system^147–150^. Importantly, YAP and TAZ act as regulators of the shape, behaviour, and function of endothelial cells during angiogenesis^151^.

**Fig. 4:**
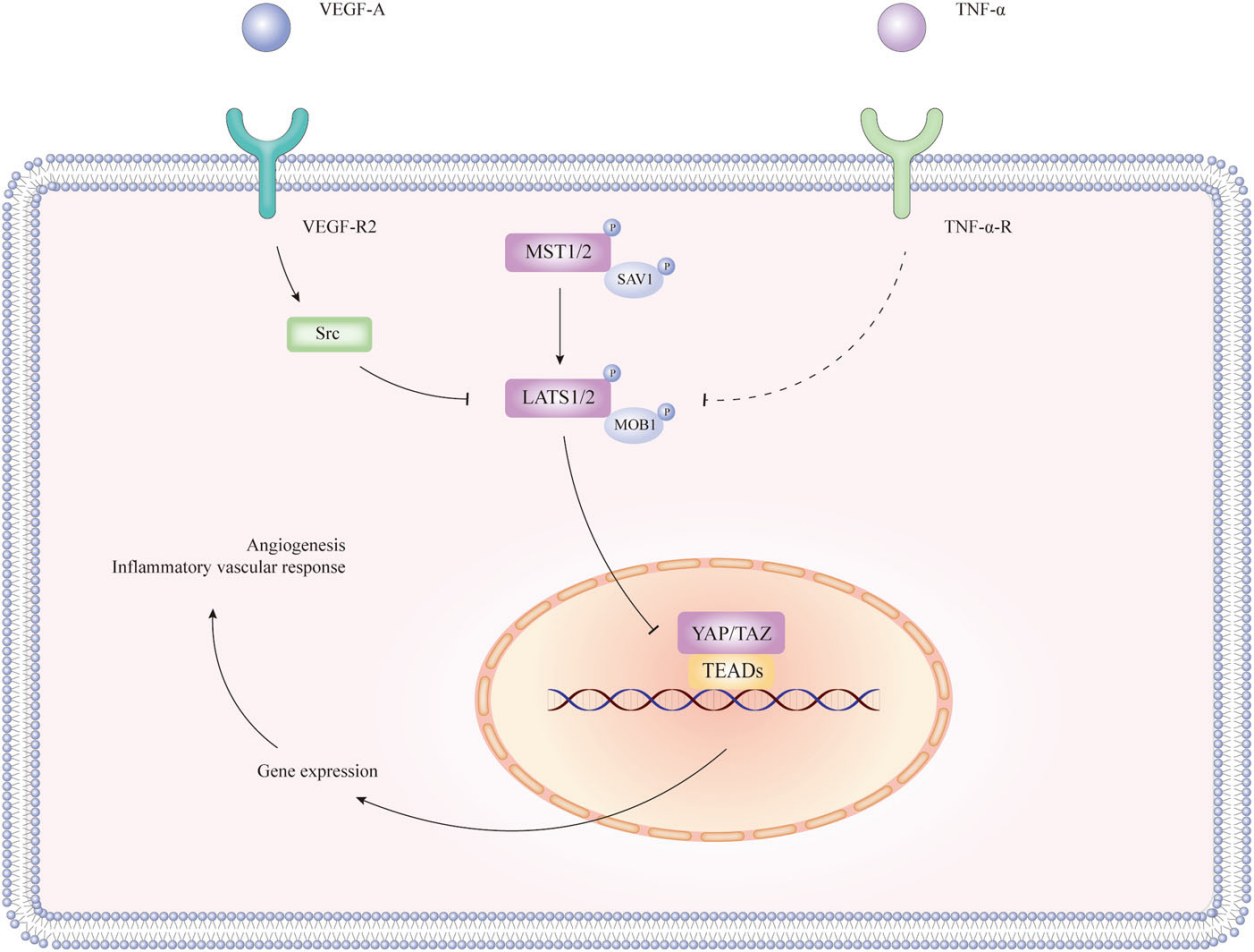
The regulation of VEGF-A and TNF-a in the Hippo pathway. VEGF-A exposure to ECs and binding with VEGFR2 may inhibit the function of LATS1/2 through Src kinase, thus relieving YAP/TAZ from inhibition and phosphorylation by LATS1/2. The complex of TNF-α and TNF-α-R may also inhibit the activity of LATS1/2 indirectly. Activated YAP/TAZ accumulates in the nucleus and promotes angiogenesis and/or inflammatory vascular response. Arrows, blunt ends, and dotted lines indicate activation, inhibition, and possible regulatory effect, respectively.

Previous studies have identified YAP/TAZ as a mediator of vascular endothelial growth factor (VEGF) signalling^152^ and a crucial regulator of angiogenesis^153^. In vitro and in vivo studies have supported that the exposure of ECs to VEGF-A inhibits the activity of LATS1/2 via binding VEGFR2 and stimulating VEGFR2-Src kinase^154^. Inactivated LATS1/2 dephosphorylates and activates YAP/TAZ, thus promoting sprouting in angiogenesis. Subsequently, Hippo signalling is activated to promote junction maturation^148,155,156^ (Fig. 4).

Notably, the tumour necrosis factor-α (TNF-α), a vascular inflammatory cytokine, has been shown to be a strong promoter of inflammation and modulator of angiogenesis^157^. TNF-α has also been suggested to inhibit the phosphorylation of LATS1/2, enhance nuclear YAP, and the increase the activity of TEADs in ECs. TNF-α requires nuclear YAP/TAZ to induce the expression of the vascular cell adhesion molecule-1 (VCAM-1) as well as intercellular adhesion molecule-1 (ICAM-1) in ECs and mediate leucocyte-endothelial adhesion, which is of great significance in inflammatory vascular responses^158^ (Fig. 4).

It is worth noting that the Hippo pathway has already been identified to function as part of pathological angiogenesis in many diseases. For example, YAP and TAZ were reported to suppress bone angiogenesis by constraining hypoxia-inducible factor signalling in ECs^159^; YAP was shown to promote angiogenesis in human cholangiocarcinoma through TEAD transcription factors^147^. Further, Hippo/YAP signalling was revealed to control the vascular remodelling in cardiovascular diseases^160^. However, the role of Hippo signalling in the pathological angiogenesis of IBD remains to be analysed and confirmed.

An anti-angiogenic treatment has been identified to treat IBD and experimental colitis models^157,161^. For example, infliximab, an anti-TNF-α monoclonal antibody, has shown promising effects in treating patients with CD^162^. Targeting the formation of new blood vessels helps to reduce tissue damage in inflammatory disorders^142^. Collectively, the Hippo pathway may serve as a target for IBD treatment.

## Discussion

The Hippo pathway is a signalling cascade that has been recently proved to play a pivotal role in regulating mammalian intestinal homoeostasis and regeneration. Generally, YAP/TAZ, the transcriptional co-activators of the Hippo pathway, are phosphorylated and inhibited by upstream kinases, including MST1/2 and LATS1/2. The activity of YAP and TAZ is the main contribution to the maintenance and proliferation of ISC and TA cells, especially when there is a tissue injury. Interestingly, IBD, a chronic and recurrent inflammatory disorder affecting the gut, requires appropriate intestinal epithelial regulation to improve the progression. YAP regulation has already been identified to regulate IBD mucosal regeneration using DSS-induced mice colitis models^8,9^. As mentioned above, knockout of YAP in mice severely dampened the intestinal regenerative capability and resulted in higher mortality rates. By contrast, wildtype mice were able to repair DSS-induced intestinal damage effectively^8^. YAP was also showed to be activated by IL-6 via gp130 upon DSS treatment to enhance intestinal epithelial cell proliferation and regeneration, whereas YAP ablation in IECs reverses DSS resistance of the gp130Act mice^9^. Furthermore, YAP/TAZ activation has been demonstrated to promote intestinal tissue repair and colonic regeneration in a mouse DSS colitis model by reprogramming the epithelium transiently into a primitive state, where the intestinal stem cells (ISCs) play an extremely important role^122^. Some studies, however, revealed the negative influence of YAP on IBD by controlling M1/M2 Macrophage Polarisation and altering gut Microbial Homoeostasis^93^. Importantly, TEADs are essential in mediating YAP-dependent gene transduction and YAP-induced cell proliferation and regeneration^19,99^, however, Hippo-YAP pathway can also regulate intestinal regeneration via the crosstalk with the WNT and Notch signals^44,98^. The function of YAP and TAZ in mammalian intestinal regeneration can be performed by TEADs as well as WNT and Notch signals, but the specific responsive mechanism of the Hippo-YAP in IBD remains unclear.

This evidence presented confirms the certain connection of the Hippo pathway and IBD, and also suggests the complicated role of Hippo-YAP in IBD. Nevertheless, there is still a lack of relevant clinical researches or reports on IBD patients by targeting the Hippo-YAP pathway. Hence the novelty and significance of this review.

Herein, we give a detailed description of the role of Hippo pathway in intestinal epithelial structure. Moreover, we also summarised the interplays of Hippo with the WNT, Notch and the role of Hippo in immune response and inflammation. Furthermore, we focused on the involvement of the Hippo pathway in IBD pathogenesis, including ISC regeneration, gut microbiota, and angiogenesis of the intestines. Collectively, several issues need to be addressed in future research and added to the collated information.

First, in view of the interplays of Hippo with the WNT, Notch, and other pathways involved in immunity and inflammation, further exploration of the roles played by those signalling pathways in the pathogenesis of IBD is imperative.

Second, it is notable that the Hippo pathway can be stimulated by tight junctions and adherence junctions. Whether targeting the Hippo pathway will influence the IEC junction structure of IBD or not deserves further investigations.

Third, due to the complex role of the Hippo pathway and crosstalk with the pathway involved in regulating the intestinal epithelial regeneration, more experiments and clinical studies need to be conducted to confirm whether targeting Hippo pathway will have implications on the patients with IBD.

Fourth, current findings have verified the role of the Hippo-YAP localised in the macrophages can alter gut microbiota. More clinical experiments are needed to identify the impact of YAP not just in macrophages but also in other cell types. In addition to YAP, whether other components of the Hippo pathway might affect the gut microbiota warrants further investigation.

Lastly, based on existing clinical and experimental studies, rather than the direct regulation of Hippo pathway, indirect regulation of Hippo pathway-associated molecules, including VEGF-A and TNF-α, has been reported to modulate angiogenesis in IBD. The VEGF-A and VEGFR-2 complex was shown to inhibit the activity of LATS1/2 of the Hippo pathway, leading to YAP/TAZ activation. VEGF-A is involved in angiogenesis observed during colitis. Levels of VEGF-A and VEGFR-2 are increased in patients with IBD and mice with colitis. In vitro experiments have shown that VEGF-A in human intestinal microvascular endothelial cells (HIMEC) may lead to angiogenic activity, while VEGF-A overexpression in vivo deteriorates DSS-induced colitis in mice^163^. Moreover, TNF-α was suggested to suppress LATS1/2. Increased TNF-α receptor levels in patients with CD and UC suggest that TNF-α receptors may regulate angiogenesis in IBD^144^. Therefore, we hypothesise that the major effectors-YAP/TAZ may promote the pathological angiogenesis in IBD via interactions with VEFG-A and VEGF-R2 as well as TNF-α, thus increasing the severity of IBD. The precise function of the Hippo pathway in the angiogenesis of IBD needs further investigation and rigorous clinical research.

A comprehensive understanding of the interplay between the Hippo pathway and IBD pathogenesis will be helpful in assessing the feasibility and efficacy of therapeutic molecules targeting Hippo pathway in IBD. Since the Hippo pathway is a signalling cascade and interfering the activity of protein kinases can be achieved by medications, developing medication that impacts the activity of upstream kinases of YAP/TAZ, including MST1/2, LATS1/2, MAP4K4, NDR1/2 may contribute to IBD treatments. Targeting YAP/TAZ or TEADs can also serve as potential therapeutic strategies for IBD. Future studies should elucidate whether patients, who suffer from IBD, will benefit from treatments regulating either the YAP/TAZ levels directly or indirectly. Activation or inhibition of certain components of the Hippo pathway, mediated by knockout or overexpression of genes associated with the Hippo pathway may alter the genesis, development, and outcomes of IBD. The use of the Hippo pathway as a therapeutic target is an attractive idea and warrants active clinical research. The results of the future studies may pave the way to diminish the morbidity and mortality associated with IBD and thus, ease the global burden of the disease.

## References

[1] Xavier RJ, Podolsky DK (2007). Unravelling the pathogenesis of inflammatory bowel disease. Nature.

[2] Kaplan GG (2015). The global burden of IBD: from 2015 to 2025. Nat. Rev. Gastroenterol. Hepatol..

[3] Ma S, Meng Z, Chen R, Guan KL (2019). The Hippo pathway: biology and pathophysiology. Annu. Rev. Biochem..

[4] Meng Z, Moroishi T, Guan KL (2016). Mechanisms of Hippo pathway regulation. Genes Dev..

[5] Zhang L (2015). NDR functions as a physiological YAP1 kinase in the intestinal epithelium. Curr. Biol..

[6] Sharif AAD, Hergovich A (2018). The NDR/LATS protein kinases in immunology and cancer biology. Semin. Cancer Biol..

[7] Meng Z (2015). MAP4K family kinases act in parallel to MST1/2 to activate LATS1/2 in the Hippo pathway. Nat. Commun..

[8] Cai J (2010). The Hippo signaling pathway restricts the oncogenic potential of an intestinal regeneration program. Genes Dev..

[9] Taniguchi K (2015). A gp130-Src-YAP module links inflammation to epithelial regeneration. Nature.

[10] Chan EH (2005). The Ste20-like kinase Mst2 activates the human large tumor suppressor kinase Lats1. Oncogene.

[11] Arranz A (2012). Akt1 and Akt2 protein kinases differentially contribute to macrophage polarization. Proc. Natl Acad. Sci. USA.

[12] Nterma P, Panopoulou E, Papadaki-Petrou E, Assimakopoulou M (2020). Immunohistochemical profile of tumor suppressor proteins RASSF1A and LATS1/2 in relation to p73 and YAP expression, of human inflammatory bowel disease and normal intestine. Pathol. Oncol. Res..

[13] Lai ZC (2005). Control of cell proliferation and apoptosis by mob as tumor suppressor, mats. Cell.

[14] Hergovich A (2016). The roles of NDR protein kinases in Hippo signalling. Genes (Basel).

[15] Wang S (2020). The crosstalk between Hippo-YAP pathway and innate immunity. Front. Immunol..

[16] Hong W, Guan KL (2012). The YAP and TAZ transcription co-activators: key downstream effectors of the mammalian Hippo pathway. Semin. Cell Dev. Biol..

[17] Lei QY (2008). TAZ promotes cell proliferation and epithelial-mesenchymal transition and is inhibited by the hippo pathway. Mol. Cell Biol..

[18] Hong AW, Meng Z, Guan KL (2016). The Hippo pathway in intestinal regeneration and disease. Nat. Rev. Gastroenterol. Hepatol..

[19] Zhao B (2008). TEAD mediates YAP-dependent gene induction and growth control. Genes Dev..

[20] Zhang H (2009). TEAD transcription factors mediate the function of TAZ in cell growth and epithelial-mesenchymal transition. J. Biol. Chem..

[21] Pan D (2010). The hippo signaling pathway in development and cancer. Dev. Cell.

[22] Chen YA (2019). WW domain-containing proteins YAP and TAZ in the Hippo pathway as key regulators in stemness maintenance, tissue homeostasis, and tumorigenesis. Front. Oncol..

[23] Harvey KF, Zhang X, Thomas DM (2013). The Hippo pathway and human cancer. Nat. Rev. Cancer.

[24] Boggiano JC, Vanderzalm PJ, Fehon RG (2011). Tao-1 phosphorylates Hippo/MST kinases to regulate the Hippo-Salvador-Warts tumor suppressor pathway. Dev. Cell.

[25] Poon CL, Lin JI, Zhang X, Harvey KF (2011). The sterile 20-like kinase Tao-1 controls tissue growth by regulating the Salvador-Warts-Hippo pathway. Dev. Cell.

[26] Vichalkovski A (2008). NDR kinase is activated by RASSF1A/MST1 in response to Fas receptor stimulation and promotes apoptosis. Curr. Biol..

[27] Oh HJ (2006). Role of the tumor suppressor RASSF1A in Mst1-mediated apoptosis. Cancer Res..

[28] Matallanas D (2007). RASSF1A elicits apoptosis through an MST2 pathway directing proapoptotic transcription by the p73 tumor suppressor protein. Mol. Cell.

[29] Yu FX (2012). Regulation of the Hippo-YAP pathway by G-protein-coupled receptor signaling. Cell.

[30] Zheng Y (2015). Identification of Happyhour/MAP4K as alternative Hpo/Mst-like kinases in the Hippo kinase cascade. Dev. Cell.

[31] Le Bouteiller M, Jensen KB (2015). Hippo signalling directs intestinal fate. Nat. Cell Biol..

[32] Ramos A, Camargo FD (2012). The Hippo signaling pathway and stem cell biology. Trends Cell Biol..

[33] Zhao B, Tumaneng K, Guan KL (2011). The Hippo pathway in organ size control, tissue regeneration and stem cell self-renewal. Nat. Cell Biol..

[34] Yu FX, Zhao B, Guan KL (2015). Hippo pathway in organ size control, tissue homeostasis, and cancer. Cell.

[35] Yu FX, Meng Z, Plouffe SW, Guan KL (2015). Hippo pathway regulation of gastrointestinal tissues. Annu. Rev. Physiol..

[36] Dong J (2007). Elucidation of a universal size-control mechanism in Drosophila and mammals. Cell.

[37] Huang J, Wu S, Barrera J, Matthews K, Pan D (2005). The Hippo signaling pathway coordinately regulates cell proliferation and apoptosis by inactivating Yorkie, the Drosophila Homolog of YAP. Cell.

[38] Guo T (2013). A novel partner of Scalloped regulates Hippo signaling via antagonizing Scalloped-Yorkie activity. Cell Res..

[39] Zhao B (2007). Inactivation of YAP oncoprotein by the Hippo pathway is involved in cell contact inhibition and tissue growth control. Genes Dev..

[40] Moroishi T (2015). A YAP/TAZ-induced feedback mechanism regulates Hippo pathway homeostasis. Genes Dev..

[41] Kodaka M, Hata Y (2015). The mammalian Hippo pathway: regulation and function of YAP1 and TAZ. Cell Mol. Life Sci..

[42] Piccolo S, Dupont S, Cordenonsi M (2014). The biology of YAP/TAZ: hippo signaling and beyond. Physiol. Rev..

[43] Deng F (2018). YAP triggers the Wnt/β-catenin signalling pathway and promotes enterocyte self-renewal, regeneration and tumorigenesis after DSS-induced injury. Cell Death Dis..

[44] Zhou D (2011). Mst1 and Mst2 protein kinases restrain intestinal stem cell proliferation and colonic tumorigenesis by inhibition of Yes-associated protein (Yap) overabundance. Proc. Natl Acad. Sci. USA.

[45] Ye S (2018). YAP1-mediated suppression of USP31 enhances NFκB activity to promote sarcomagenesis. Cancer Res..

[46] Lee IY (2019). MST1 negatively regulates TNFα-induced NF-κB signaling through modulating LUBAC activity. Mol. Cell.

[47] Yu M (2018). MicroRNA-590-5p inhibits intestinal inflammation by targeting YAP. J. Crohns Colitis.

[48] Itoh K, Brott BK, Bae GU, Ratcliffe MJ, Sokol SY (2005). Nuclear localization is required for Dishevelled function in Wnt/beta-catenin signaling. J. Biol..

[49] van der Flier LG, Clevers H (2009). Stem cells, self-renewal, and differentiation in the intestinal epithelium. Annu. Rev. Physiol..

[50] Batlle E (2002). Beta-catenin and TCF mediate cell positioning in the intestinal epithelium by controlling the expression of EphB/ephrinB. Cell.

[51] Korinek V (1998). Depletion of epithelial stem-cell compartments in the small intestine of mice lacking Tcf-4. Nat. Genet.

[52] Imajo M, Miyatake K, Iimura A, Miyamoto A, Nishida E (2012). A molecular mechanism that links Hippo signalling to the inhibition of Wnt/β-catenin signalling. Embo J..

[53] Varelas X (2010). The Hippo pathway regulates Wnt/beta-catenin signaling. Dev. Cell.

[54] Rosenbluh J (2012). β-Catenin-driven cancers require a YAP1 transcriptional complex for survival and tumorigenesis. Cell.

[55] Heallen T (2011). Hippo pathway inhibits Wnt signaling to restrain cardiomyocyte proliferation and heart size. Science.

[56] Konsavage WM, Kyler SL, Rennoll SA, Jin G, Yochum GS (2012). Wnt/β-catenin signaling regulates Yes-associated protein (YAP) gene expression in colorectal carcinoma cells. J. Biol. Chem..

[57] Azzolin L (2012). Role of TAZ as mediator of Wnt signaling. Cell.

[58] Kopan R, Ilagan MX (2009). The canonical Notch signaling pathway: unfolding the activation mechanism. Cell.

[59] Bray SJ (2006). Notch signalling: a simple pathway becomes complex. Nat. Rev. Mol. Cell Biol..

[60] Camargo FD (2007). YAP1 increases organ size and expands undifferentiated progenitor cells. Curr. Biol..

[61] Lawrence T (2009). The nuclear factor NF-kappaB pathway in inflammation. Cold Spring Harb. Perspect. Biol..

[62] Sun SC (2017). The non-canonical NF-κB pathway in immunity and inflammation. Nat. Rev. Immunol..

[63] Karin M, Ben-Neriah Y (2000). Phosphorylation meets ubiquitination: the control of NF-[kappa]B activity. Annu. Rev. Immunol..

[64] Vallabhapurapu S, Karin M (2009). Regulation and function of NF-kappaB transcription factors in the immune system. Annu. Rev. Immunol..

[65] Deng Y (2018). Reciprocal inhibition of YAP/TAZ and NF-κB regulates osteoarthritic cartilage degradation. Nat. Commun..

[66] Lv Y (2018). YAP controls endothelial activation and vascular inflammation through TRAF6. Circ. Res.

[67] Wang Q (2018). REGγ controls Hippo signaling and reciprocal NF-κB-YAP regulation to promote colon cancer. Clin. Cancer Res..

[68] Zhou Y (2018). Emerging roles of Hippo signaling in inflammation and YAP-driven tumor immunity. Cancer Lett..

[69] Medzhitov R (2008). Origin and physiological roles of inflammation. Nature.

[70] Ben-Neriah Y, Karin M (2011). Inflammation meets cancer, with NF-κB as the matchmaker. Nat. Immunol..

[71] Liu B (2016). Toll receptor-mediated hippo signaling controls innate immunity in drosophila. Cell.

[72] Zhang Q (2018). Yes-associated protein (YAP) and transcriptional coactivator with PDZ-binding motif (TAZ) mediate cell density-dependent proinflammatory responses. J. Biol. Chem..

[73] LaCanna R (2019). Yap/Taz regulate alveolar regeneration and resolution of lung inflammation. J. Clin. Invest.

[74] Hagenbeek TJ (2018). The Hippo pathway effector TAZ induces TEAD-dependent liver inflammation and tumors. Sci. Signal..

[75] Ye X, Ong N, An H, Zheng Y (2020). The emerging roles of NDR1/2 in infection and inflammation. Front. Immunol..

[76] Song H, Wang M, Xin T (2019). Mst1 contributes to nasal epithelium inflammation via augmenting oxidative stress and mitochondrial dysfunction in a manner dependent on Nrf2 inhibition. J. Cell Physiol..

[77] Garbers C (2012). Plasticity and cross-talk of interleukin 6-type cytokines. Cytokine Growth Factor Rev..

[78] Moroishi T (2016). The Hippo pathway kinases LATS1/2 suppress cancer immunity. Cell.

[79] Tang F (2015). The kinases NDR1/2 act downstream of the Hippo homolog MST1 to mediate both egress of thymocytes from the thymus and lymphocyte motility. Sci. Signal.

[80] Li J (2015). Mammalian sterile 20-like kinase 1 (Mst1) enhances the stability of forkhead box P3 (Foxp3) and the function of regulatory T cells by modulating Foxp3 acetylation. J. Biol. Chem..

[81] Du X (2014). Mst1/Mst2 regulate development and function of regulatory T cells through modulation of Foxo1/Foxo3 stability in autoimmune disease. J. Immunol..

[82] Nehme NT (2012). MST1 mutations in autosomal recessive primary immunodeficiency characterized by defective naive T-cell survival. Blood.

[83] Abdollahpour H (2012). The phenotype of human STK4 deficiency. Blood.

[84] Geng J (2017). The transcriptional coactivator TAZ regulates reciprocal differentiation of T(H)17 cells and T(reg) cells. Nat. Immunol..

[85] Geremia A, Biancheri P, Allan P, Corazza GR, Di Sabatino A (2014). Innate and adaptive immunity in inflammatory bowel disease. Autoimmun. Rev..

[86] Zhou L (2007). IL-6 programs T(H)-17 cell differentiation by promoting sequential engagement of the IL-21 and IL-23 pathways. Nat. Immunol..

[87] Fujino S (2003). Increased expression of interleukin 17 in inflammatory bowel disease. Gut.

[88] Rovedatti L (2009). Differential regulation of interleukin 17 and interferon gamma production in inflammatory bowel disease. Gut.

[89] Fantini MC (2006). Transforming growth factor beta induced FoxP3+ regulatory T cells suppress Th1 mediated experimental colitis. Gut.

[90] Singh B (2001). Control of intestinal inflammation by regulatory T cells. Immunol. Rev..

[91] Lawrence T, Natoli G (2011). Transcriptional regulation of macrophage polarization: enabling diversity with identity. Nat. Rev. Immunol..

[92] Kühl AA, Erben U, Kredel LI, Siegmund B (2015). Diversity of intestinal macrophages in inflammatory bowel diseases. Front. Immunol..

[93] Zhou X (2019). YAP aggravates inflammatory bowel disease by regulating M1/M2 macrophage polarization and gut microbial homeostasis. Cell Rep..

[94] Gehart H, Clevers H (2019). Tales from the crypt: new insights into intestinal stem cells. Nat. Rev. Gastroenterol. Hepatol..

[95] Crosnier C, Stamataki D, Lewis J (2006). Organizing cell renewal in the intestine: stem cells, signals and combinatorial control. Nat. Rev. Genet.

[96] Sato T (2011). Paneth cells constitute the niche for Lgr5 stem cells in intestinal crypts. Nature.

[97] Sasaki N (2016). Reg4+ deep crypt secretory cells function as epithelial niche for Lgr5+ stem cells in colon. Proc. Natl Acad. Sci. USA.

[98] Barry ER (2013). Restriction of intestinal stem cell expansion and the regenerative response by YAP. Nature.

[99] Imajo M, Ebisuya M, Nishida E (2015). Dual role of YAP and TAZ in renewal of the intestinal epithelium. Nat. Cell Biol..

[100] Sun S, Irvine KD (2016). Cellular organization and cytoskeletal regulation of the hippo signaling network. Trends Cell Biol..

[101] Schulzke JD (2009). Epithelial tight junctions in intestinal inflammation. Ann. N. Y. Acad. Sci..

[102] Kim NG, Koh E, Chen X, Gumbiner BM (2011). E-cadherin mediates contact inhibition of proliferation through Hippo signaling-pathway components. Proc. Natl Acad. Sci. USA.

[103] Chen L, Qin F, Deng X, Avruch J, Zhou D (2012). Hippo pathway in intestinal homeostasis and tumorigenesis. Protein Cell.

[104] Ananthakrishnan AN (2015). Epidemiology and risk factors for IBD. Nat. Rev. Gastroenterol. Hepatol..

[105] Lin Y (2014). Chemerin aggravates DSS-induced colitis by suppressing M2 macrophage polarization. Cell. Mol. Immunol..

[106] Ramos GP, Papadakis KA (2019). Mechanisms of disease: inflammatory bowel diseases. Mayo Clin. Proc..

[107] Perse M, Cerar A (2012). Dextran sodium sulphate colitis mouse model: traps and tricks. J. Biomed. Biotechnol..

[108] Elson CO, Sartor RB, Tennyson GS, Riddell RH (1995). Experimental models of inflammatory bowel disease. Gastroenterology.

[109] Neurath M, Fuss I, Strober W (2000). TNBS-colitis. Int. Rev. Immunol..

[110] Okayasu I (1990). A novel method in the induction of reliable experimental acute and chronic ulcerative colitis in mice. Gastroenterology.

[111] Farooq SM (2009). Therapeutic effect of blocking CXCR2 on neutrophil recruitment and dextran sodium sulfate-induced colitis. J. Pharmacol. Exp. Ther..

[112] Berndt BE, Zhang M, Chen GH, Huffnagle GB, Kao JY (2007). The role of dendritic cells in the development of acute dextran sulfate sodium colitis. J. Immunol..

[113] Antoniou E (2016). The TNBS-induced colitis animal model: An overview. Ann. Med. Surg. (Lond.).

[114] Mizoguchi A (2012). Animal models of inflammatory bowel disease. Prog. Mol. Biol. Transl. Sci..

[115] Morris GP (1989). Hapten-induced model of chronic inflammation and ulceration in the rat colon. Gastroenterology.

[116] Kurashima Y, Kiyono H (2017). Mucosal ecological network of epithelium and immune cells for gut homeostasis and tissue healing. Annu. Rev. Immunol..

[117] Cader MZ, Kaser A (2013). Recent advances in inflammatory bowel disease: mucosal immune cells in intestinal inflammation. Gut.

[118] Odenwald MA, Turner JR (2017). The intestinal epithelial barrier: a therapeutic target?. Nat. Rev. Gastroenterol. Hepatol..

[119] Neurath MF, Travis SP (2012). Mucosal healing in inflammatory bowel diseases: a systematic review. Gut.

[120] Okamoto R, Watanabe M (2016). Role of epithelial cells in the pathogenesis and treatment of inflammatory bowel disease. J. Gastroenterol..

[121] Azzolin L (2014). YAP/TAZ incorporation in the β-catenin destruction complex orchestrates the Wnt response. Cell.

[122] Yui S (2018). YAP/TAZ-dependent reprogramming of colonic epithelium links ECM remodeling to tissue regeneration. Cell Stem Cell.

[123] Mahoney JE, Mori M, Szymaniak AD, Varelas X, Cardoso WV (2014). The hippo pathway effector Yap controls patterning and differentiation of airway epithelial progenitors. Dev. Cell.

[124] Gregorieff A, Liu Y, Inanlou MR, Khomchuk Y, Wrana JL (2015). Yap-dependent reprogramming of Lgr5(+) stem cells drives intestinal regeneration and cancer. Nature.

[125] Jarriault S (1995). Signalling downstream of activated mammalian Notch. Nature.

[126] Jeon MK, Klaus C, Kaemmerer E, Gassler N (2013). Intestinal barrier: Molecular pathways and modifiers. World J. Gastrointest. Pathophysiol..

[127] Okamoto R (2009). Requirement of Notch activation during regeneration of the intestinal epithelia. Am. J. Physiol. Gastrointest. Liver Physiol..

[128] Sancho R, Cremona CA, Behrens A (2015). Stem cell and progenitor fate in the mammalian intestine: Notch and lateral inhibition in homeostasis and disease. EMBO Rep..

[129] van Es JH (2005). Notch/gamma-secretase inhibition turns proliferative cells in intestinal crypts and adenomas into goblet cells. Nature.

[130] Grivennikov S (2009). IL-6 and Stat3 are required for survival of intestinal epithelial cells and development of colitis-associated cancer. Cancer Cell.

[131] Ernst M, Thiem S, Nguyen PM, Eissmann M, Putoczki TL (2014). Epithelial gp130/Stat3 functions: an intestinal signaling node in health and disease. Semin. Immunol..

[132] Nishida A (2018). Gut microbiota in the pathogenesis of inflammatory bowel disease. Clin. J. Gastroenterol..

[133] Ni J, Wu GD, Albenberg L, Tomov VT (2017). Gut microbiota and IBD: causation or correlation?. Nat. Rev. Gastroenterol. Hepatol..

[134] Mosca A, Leclerc M, Hugot JP (2016). Gut microbiota diversity and human diseases: should we reintroduce key predators in our ecosystem?. Front. Microbiol..

[135] Ott SJ (2004). Reduction in diversity of the colonic mucosa associated bacterial microflora in patients with active inflammatory bowel disease. Gut.

[136] Manichanh C (2006). Reduced diversity of faecal microbiota in Crohn’s disease revealed by a metagenomic approach. Gut.

[137] Franzosa EA (2019). Gut microbiome structure and metabolic activity in inflammatory bowel disease. Nat. Microbiol.

[138] Hold GL (2014). Role of the gut microbiota in inflammatory bowel disease pathogenesis: what have we learnt in the past 10 years?. World J. Gastroenterol..

[139] Wang W (2014). Increased proportions of Bifidobacterium and the Lactobacillus group and loss of butyrate-producing bacteria in inflammatory bowel disease. J. Clin. Microbiol..

[140] Matsuoka K, Kanai T (2015). The gut microbiota and inflammatory bowel disease. Semin. Immunopathol..

[141] Sajib S, Zahra FT, Lionakis MS, German NA, Mikelis CM (2018). Mechanisms of angiogenesis in microbe-regulated inflammatory and neoplastic conditions. Angiogenesis.

[142] Danese S (2007). Angiogenesis blockade as a new therapeutic approach to experimental colitis. Gut.

[143] Danese S (2006). Angiogenesis as a novel component of inflammatory bowel disease pathogenesis. Gastroenterology.

[144] Chidlow JH (2006). Differential angiogenic regulation of experimental colitis. Am. J. Pathol..

[145] Hatoum OA, Binion DG (2005). The vasculature and inflammatory bowel disease: contribution to pathogenesis and clinical pathology. Inflamm. Bowel Dis..

[146] Boopathy GTK, Hong W (2019). Role of Hippo pathway-YAP/TAZ signaling in angiogenesis. Front. Cell Dev. Biol..

[147] Marti P (2015). YAP promotes proliferation, chemoresistance, and angiogenesis in human cholangiocarcinoma through TEAD transcription factors. Hepatology.

[148] Choi HJ (2015). Yes-associated protein regulates endothelial cell contact-mediated expression of angiopoietin-2. Nat. Commun..

[149] Yuan L (2017). Palmitic acid dysregulates the Hippo-YAP pathway and inhibits angiogenesis by inducing mitochondrial damage and activating the cytosolic DNA sensor cGAS-STING-IRF3 signaling mechanism. J. Biol. Chem..

[150] Kim J (2017). YAP/TAZ regulates sprouting angiogenesis and vascular barrier maturation. J. Clin. Invest.

[151] Azad T, Ghahremani M, Yang X (2019). The role of YAP and TAZ in angiogenesis and vascular mimicry. Cells.

[152] Wang X (2017). YAP/TAZ orchestrate VEGF signaling during developmental angiogenesis. Dev. Cell.

[153] Azad T (2018). A LATS biosensor screen identifies VEGFR as a regulator of the Hippo pathway in angiogenesis. Nat. Commun..

[154] Park JA, Kwon YG (2018). Hippo-YAP/TAZ signaling in angiogenesis. BMB Rep..

[155] Choi HJ, Kwon YG (2015). Roles of YAP in mediating endothelial cell junctional stability and vascular remodeling. BMB Rep..

[156] He J (2018). Yes-associated protein promotes angiogenesis via signal transducer and activator of transcription 3 in endothelial cells. Circ. Res.

[157] Chidlow JH, Shukla D, Grisham MB, Kevil CG (2007). Pathogenic angiogenesis in IBD and experimental colitis: new ideas and therapeutic avenues. Am. J. Physiol. Gastrointest. Liver Physiol..

[158] Choi HJ, Kim NE, Kim BM, Seo M, Heo JH (2018). TNF-α-induced YAP/TAZ activity mediates leukocyte-endothelial adhesion by regulating VCAM1 expression in endothelial cells. Int. J. Mol. Sci..

[159] Sivaraj KK (2020). YAP1 and TAZ negatively control bone angiogenesis by limiting hypoxia-inducible factor signaling in endothelial cells. Elife.

[160] He J (2018). The role of Hippo/yes-associated protein signalling in vascular remodelling associated with cardiovascular disease. Br. J. Pharm..

[161] Sandor Z, Deng XM, Khomenko T, Tarnawski AS, Szabo S (2006). Altered angiogenic balance in ulcerative colitis: a key to impaired healing?. Biochem. Biophys. Res. Commun..

[162] Stokkers PC, Hommes DW (2004). New cytokine therapeutics for inflammatory bowel disease. Cytokine.

[163] Scaldaferri F (2009). VEGF-A links angiogenesis and inflammation in inflammatory bowel disease pathogenesis. Gastroenterology.

